# Interprofessional Curriculum Delivery: Experience of a Primary Care Education Program

**DOI:** 10.3390/healthcare12090950

**Published:** 2024-05-06

**Authors:** Jessica A. Davila, Nancy D. Harada, Kathryn Wirtz Rugen, Stuart C. Gilman, Shubhada Sansgiry

**Affiliations:** 1 Center for Innovations in Quality, Effectiveness and Safety, Michael E. DeBakey Veterans Affairs Medical Center, 2002 Holcombe Blvd. (MS152), Houston, TX 77030, USA; shubhada.sansgiry@va.gov; 2 Department of Medicine, Baylor College of Medicine, Houston, TX 77030, USA; 3 Office of Academic Affiliations, Department of Veterans Affairs, 810 Vermont Ave NW, Washington, DC 20420, USA; nancy.harada@va.gov (N.D.H.); scgilman@gmail.com (S.C.G.); 4 Office of Nursing Services, Department of Veterans Affairs, 810 Vermont Ave NW, Washington, DC 20420, USA; kathryn.rugen@va.gov; 5 College of Nursing, University of Illinois at Chicago, 845 S Damen Ave MC 802, Chicago, IL 60612, USA; 6 Veterans Affairs South Central Mental Illness Research Education and Clinical Center, 2002 Holcombe Blvd. (MS152), Houston, TX 77030, USA

**Keywords:** interprofessional education, health profession HPTs, team based, health professions, program evaluation

## Abstract

Few post-graduate training programs offer a comprehensive curriculum that includes structured clinical experiences to teach interprofessional care. To address this need, the United States Department of Veterans Affairs, Office of Academic Affiliations funded the Centers of Excellence in Primary Care Education (CoEPCE) from 2011–2019 to provide interprofessional curricula for health profession trainees (HPTs), including physician residents, nurse practitioner residents, pharmacy residents, and psychology residents. We examined changes over time in curricular domains, system impacts, and program practices based on HPT survey data and the qualitative evaluation of narrative feedback. An annual survey was administered to participants. Indirect standardized ratios were calculated for interprofessional professional education (IPE) program domains, system impacts, and program practices. Qualitative responses were coded based on curricular domains and key program components. The study cohort included 369 HPTs. Site and profession standardized indirect ratios across all professions indicated improvements in curricular domains, system impacts, and program practices, with significant differences observed for associated health HPTs as compared to other HPTs for performance improvement. Qualitative data indicated that profession was associated with differences in perceptions of the curriculum. Although improvements occurred over time, our findings support the need for the thoughtful consideration of profession-specific identity characteristics when designing interprofessional curricula.

## 1. Introduction

Interprofessional training has been shown to significantly improve communication and collaboration among healthcare providers from different professions [1,2,3,4,5], as well as to positively impact the delivery of patient care [6]. In the primary care setting, interprofessional training is critical; modern primary care practice emphasizes the importance of a team-based, multidisciplinary approach to properly address the needs of patients with multiple comorbidities in the context of adverse psychosocial and environmental issues [7]. Yet, there has been insufficient emphasis on interprofessional practice in health profession training for primary care professions, and few post-graduate interprofessional training programs have focused exclusively on the primary care setting [8]. There is a need for comprehensive interprofessional curricula specifically for primary care that includes didactic learning, hands-on clinical experiences, and dedicated mentoring to teach providers from different professions how to successfully work together to manage complex patients in an outpatient environment. The Department of Veterans Affairs Centers of Excellence in Primary Care Education (CoEPCE) was one such program funded by the VA Office of Academic Affiliations (OAA), with the purpose of developing and implementing a primary care-focused curricula that included specialized interprofessional education training based on profession, the cross-collaborative mentoring of health professions trainees (HPTs) by faculty from various professions, and community-focused clinical experiences to learn how to effectively deliver interprofessional care [9].

CoEPCE programs were located at seven geographically diverse VA facilities across the US, with the overall mission of teaching HPTs, with an interest in primary care to delivering high-quality, interprofessional, patient-centered care. HPTs included physician residents, nurse practitioner residents, pharmacy residents, and psychology residents. A foundational common curriculum focused on achieving learning outcomes related to the four core interprofessional professional education (IPE) program domains (interprofessional collaboration, shared decision making, sustained relationships, and performance improvement) was implemented across all centers [9]. CoEPCE programs worked individually and collectively to develop teaching strategies aligned with the four core program domains to achieve common program and learning outcomes across HPT professions.

Over time, the CoEPCE program was refined to better educate HPTs on the four curricular domains, expand the program to accommodate the learning needs of HPTs from additional disciplines, and address local facility and community differences. Other studies have examined the impacts of the clinical outcomes associated with the CoECPE interventions, demonstrating at least noninferiority and, in some cases, patient improvements when compared to care provided in other training and staff-only primary care settings [10,11,12]. Perceptions of staff participating in the CoEPCE have also been described [13]. In order to understand possible mechanisms for the effectiveness of such innovative curricula in primary care education, it is essential to pursue qualitative analyses of HPT perceptions, including examining the potential impact of the trainee’s professions. In this study, we analyze trends over time in HPT perceptions of the CoEPCE program across the four core program domains and key program elements by profession through quantitative and qualitative analyses of surveys of HPTs who participated in the CoEPCE.

## 2. Materials and Methods

### 2.1. CoEPCE Curriculum

**Figure 1:**
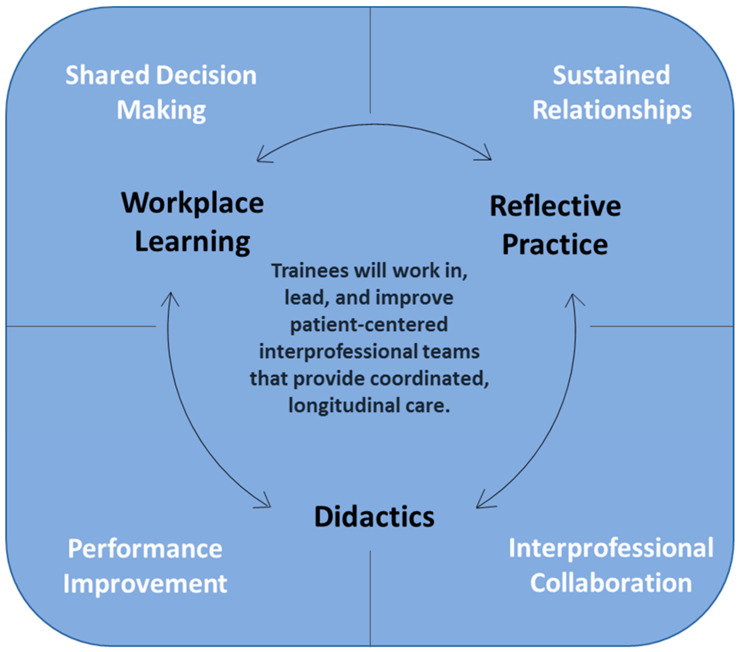
Four core curricular domains and instructional approaches for Centers of Excellence in Primary Care Education.

At each of the seven VA CoEPCE sites, local interprofessional curricula was developed and delivered according to the national VA CoEPCE program framework presented in Figure 1 [14]. A variety of instructional approaches (didactics, workplace learning, and reflective practice) [9] were used to address the four core domains: interprofessional collaboration, sustained relationships, shared decision making, and performance improvement. These educational domains and the implementation of curricula have been described in several previously published studies [9,15]. In brief, interprofessional collaboration involved trustful, collaborative relationships among professions for delivering team-based, coordinated care; sustained relationships included fostering respectful and trusting relationships between patients, families, and other health professionals; shared decision making involved supporting patients to make healthcare decisions that embraced their values and preferences; and performance improvement trained HPTs to foster a culture of continuous improvement and assessment to optimize patient outcomes. These domains were consistent with other interprofessional education (IPE) programs [13].

Unlike other specialized IPE programs, the VA CoEPCE program simultaneously delivered a standardized IPE curriculum across sites, in addition to unique local curricula from multiple professions at each site. The curriculum to teach trainees interprofessional skills at all sites included both classroom learning, as well as clinical experiences under the guidance of a faculty mentor. Local variation in the curriculum was determined based primarily on geographical differences and the needs of the patient population. Trainees could participate in the CoEPE program for one to two years, depending on the profession and training preferences. Nurse practitioner residents often completed two years of the program, whereas other HPTs only participated for one year.

The CoEPCE program was implemented in the primary care clinic setting. HPTs could apply IPE skills in a real-world setting with HPTs from other professions under faculty supervision and receive feedback. All HPTs practiced common program components in the clinical setting, yet profession-specific training experiences were offered based on prior experiences of HPTs, accreditation requirements, and the length of training.

### 2.2. CoEPCE Health Professions Trainees Participant Survey

An interprofessional evaluation team, including VA CoEPCE evaluators and an external VA research group, developed the annual survey. This survey was used for program evaluation purposes rather than individual HPT assessment. The goal of the survey was to examine differences in the perceptions of the CoEPCE program and identify unmet needs by profession. Survey results were used to make programmatic changes in order to better meet HPT needs and expectations. A previous study was published describing in detail the development and validation of this survey [14]. The final survey instrument included a total of 24 questions, with some questions containing multiple items.

This survey and the associated analyses are categorized as an operation’s improvement activity based on the VHA Handbook 1200.21, where the information generated is used for business operations and quality improvement. The overall project was subject to administrative oversight rather than oversight from a Human Subjects Institutional Review Board.

### 2.3. Data Collection Procedures

Beginning in the academic year (AY) 2016–2017, this cross-sectional survey was administered annually via SurveyMonkey to enrolled HPTs from the seven CoEPCE sites (Boise, Cleveland, Greater Los Angeles, Houston, San Francisco, Seattle, and West Haven). Invitations to participate were emailed to HPTs and included a direct link to the survey. In addition, CoEPCE site directors sent personalized, follow-up emails requesting HPTs to complete the survey. Responses were monitored and email reminders were sent approximately every two weeks for four weeks to those HPTs who had not responded. Identifiers were not linked with survey responses. Between AY16-17 through to AY18-19, the survey response rates ranged from 43% to 58%.

### 2.4. Survey Items

Information on HPT professions, training sites, time spent in the program, and the year of training were collected. Two open-ended items were also included at the end of the survey, where participants could provide feedback on the strengths of the program, as well as areas for improvement.

### 2.5. Core CoEPCE Program Domain Subscales

Interprofessional collaboration was measured via the amount of interprofessional communication practiced by HPTs as part of delivering clinical care during their training using a 4-item, 5-point Likert scale, ranging from 1 (never) to 5 (always).

Shared decision making was measured via the how much HPTs engaged in shared decision making, including communicating with patients, using motivational interviewing techniques, and utilizing telehealth using a 5-item, 5-point Likert scale, ranging from 1 (never) to 5 (always).

Sustained relationships were measured via the frequency with which HPTs engaged in and navigated relationships with patients and other health professionals based on a 4-item, 5-point Likert scale, ranging from 1 (never) to 5 (always).

Performance improvement was measured via the use of skills related to evaluation and improvement of clinical practice based on a 4-item, 5-point Likert scale, ranging from 1 (never) to 5 (always).

### 2.6. Key CoEPCE Program Elements Subscales

*System impacts* measured contributions of the CoEPCE program to improving the care provided by local VA facilities and enhancing the educational environment, including supporting best practices in primary care. This also included fostering relationships with academic affiliates. This domain was assessed based on respondents’ agreement with statements about the relationship with the VA CoEPCE program and their local facility, and consisted of a 9-item, 5-point Likert scale, ranging from 1 (strongly disagree) to 5 (strongly agree).

Program practices focused on HPTs’ learning experiences during their training program, including understanding expectations and receiving clear feedback on their performance, access to interprofessional learning opportunities, and mentorship in achieving career goals. This domain consisted of a 7-item, 5-point Likert scale, ranging from 1 (strongly disagree) to 5 (strongly agree).

### 2.7. Data Analysis

#### 2.7.1. Quantitative Analyses

Data were analyzed using SAS^®^ 9.4 (SAS Institute, Inc., Cary, NC, USA). Descriptive statistics were calculated for HPT characteristics. Missing values were imputed using the mean value by AY, site, and profession. The average score of items included in each of the four core domains, program practices, and system impacts were calculated for each HPT, and were subsequently dichotomized into a score of <4 or ≥4. Indirect standardization methods were then used to compare scores across AYs, with 2016–2017 as the comparison or base year (AY16-17). Indirect standardization methods were used, given the small sample within each AY. Using the dichotomized scores, observed to expected ratios were calculated and standardized by site and profession. These ratios were then compared to the baseline AY16-17 for the four core domains, system impacts, and program practices. Confidence intervals (95%) were calculated using the exact Poisson method [16].

#### 2.7.2. Qualitative Analyses

We used rapid qualitative analysis methods to evaluate responses from the two open-ended questions to further explore HPTs perceptions of CoEPCE over time and across sites [17,18]. Study members with experience in rapid analysis (JD, NH) worked together to code individual comments by domain, create domain summaries, and identify main themes. The resulting domain summaries and themes were then reviewed by other members of the study team to ensure validity.

## 3. Results

Our study cohort consisted of 369 HPT respondents across the three years as follows: AY16-17 (36.6%), AY17-18 (34.9%), and AY18-19 (28.5%). The sample was comprised of 45.8% physician residents, 28.5% nurse practitioner HPTs, and 25.7% associated health professionals (i.e., pharmacy residents, psychology fellows) across the three-year study period. Table 1 shows the mean and median values by profession at the baseline. There were no significant differences in the median values, except for system impacts.

### 3.1. Indirect Standardization of Domain Scores

**Figure 2:**
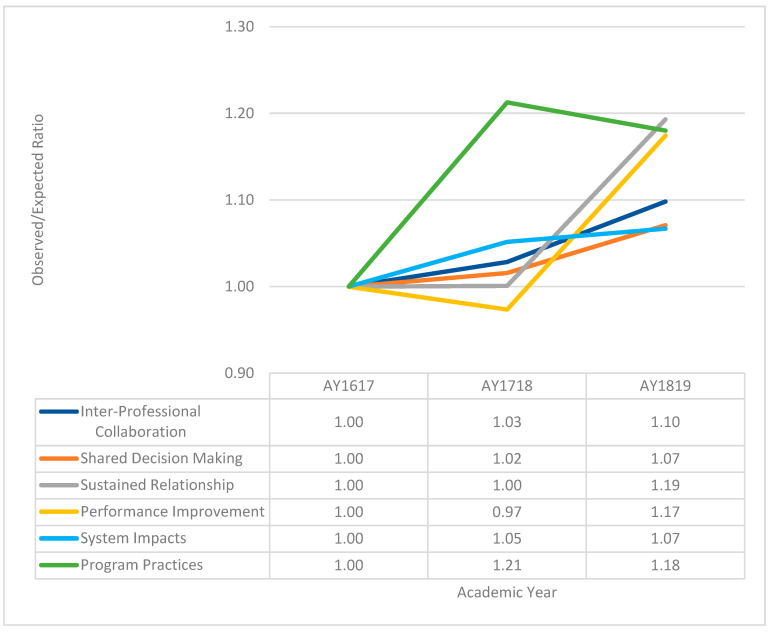
Trends over time in the ratio of observed to expected responses by curricular domain for all professions combined using indirect standardization. The baseline academic year is 2016–2017. The confidence intervals calculated for the observed to expected ratios for the four core domains, system impacts, and program practices were not significantly different over time.

Figure 2 shows the site and profession standardized rates (observed/expected ratios) to base AY16-17 for AY17-18 and AY18-19. There were no significant differences in mean values at the baseline, except for system impact. This graph shows how each domain changed from the base year (AY16-17) in subsequent AYs among all HPTs. Specifically, interprofessional collaboration improved by 3% in AY17-18 and 10% in AY18-19. Shared decision making improved by 2% in AY17-18 and 7% in AY18-19. There was no change in sustained relationships in AY17-18, and a 19% improvement was observed in AY18-19. The performance improvement declined by 3% in AY17-18, but improved by 17% in AY18-19. The program practices improved by 21% in AY17-18 and 18% in AY18-19. The system impact showed only slight improvements of 5% (AY17-18) and 7% (AY18-19). Across all professions, HPTs perceived improvement over the base year in the site and profession standardized indirect ratios for curricular domains, system impacts, and program practices, but the increase was not statistically significant.

**Figure 3:**
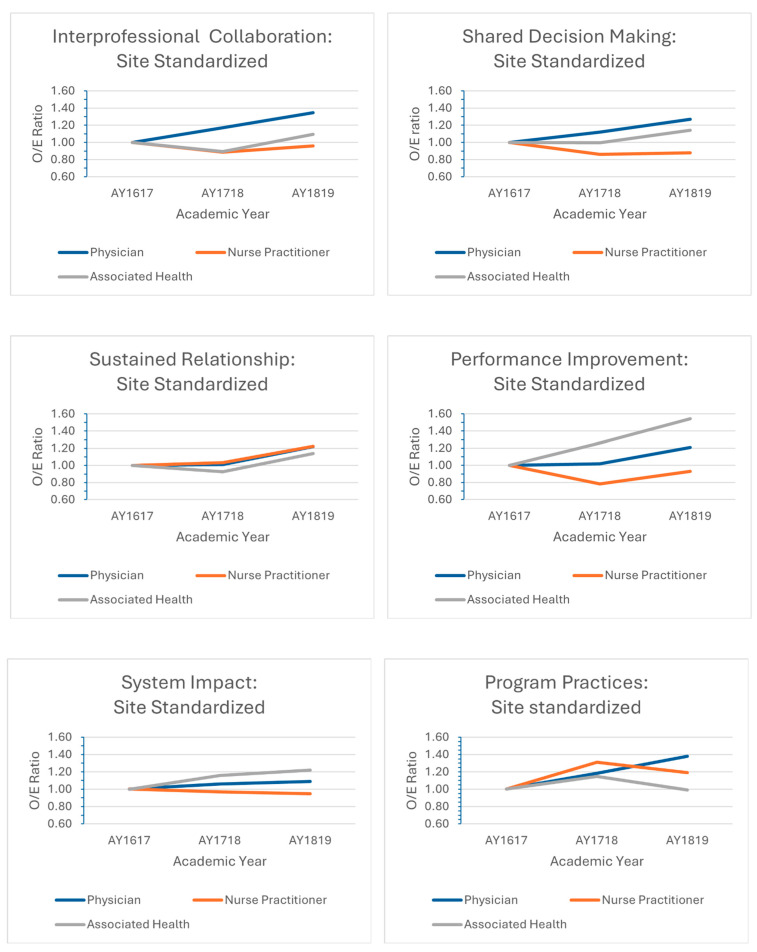
Trends over time in the ratio of observed to expected responses by curricular domain stratified by profession using indirect standardization. The baseline academic year is 2016–2017.

Figure 3 represents the change from the base year in the observed to expected ratios by profession, which were standardized by site. Interprofessional collaboration improved over time as follows: 17% for AY17-18 and 35% for AY18-19 for physician residents. For nurse practitioner HPTs, interprofessional collaboration was lower (−11% for AY17-18 and −4% for AY18-19). For associated health HPTs, interprofessional collaboration was lower (−11%) for AY17-18, but improved by 9% for AY18-19.

Shared decision making indicated similar changes over time, with physician residents showing an improvement (12% for AY18-17; 27% for AY18-19). Nurse practitioner HPTs showed a decline (−14% for AY17-18; and −12% for AY18-19), and associated health HPTs were stable for AY17-18, but improved by 14% for AY18-19.

Sustained relationships showed a slight improvement for physician residents (1%) and nurse practitioner HPTs (3%) for AY17-18, but declined among associated health HPTs (−7% for AY17-18). Improvement within all three professions were seen for AY18-19 with a 22% improvement for both physician residents and nurse practitioner HPTs, and a 14% improvement for associated health HPTs.

A 26% increase in performance improvement among associated health HPTs was observed for AY17-18, and a 54% increase for AY18-19. Physician residents also showed a slight improvement of 2% for AY17-18 and 21% for AY18-19. Nurse practitioner HPTs showed declines during both time periods (−22% for AY17-18 and −7% for AY18-19).

Program practices improved for physician residents (18% for AY17-18 and 38% for AY18-19) and nursing HPTs (31% for AY17-18 and 19% for AY18-19). Associated health HPTs improved by 15% for AY17-18, but then slightly declined by 1% for AY18-19.

The system impact improved only slightly for physician residents (6% for AY17-18 and 9% for AY18-19), declined slightly for nurse practitioner HPTs (−3% for AY17-18 and −5% for AY18-19), and improved for associated health HPTs (16% for AY17-18 and 22% for AY18-19).

Though the performance improvement indirect standardized ratio by site for the Associated Health group was significantly higher at AY18-19 when compared with the base year (1.54 (95% CI 1.03–2.24)), all others curricular domains, system impacts, and program practices did not indicate statistical significance.

### 3.2. Qualitative Rapid Analysis

The domains and themes identified from the rapid analysis of the qualitative data are displayed in Table 2. Domains included (1) shared decision making; (2) sustained relationships; (3) interprofessional collaboration; (4) performance improvement; (5) clinical knowledge and competence; (6) program structure; and (7) professional development.

Most comments were regarding interprofessional collaboration (n = 418), program structure (n = 325), and clinical knowledge and competence (n = 227). Prominent themes related to interprofessional collaboration were understanding the backgrounds of other health professionals, the challenges of working as part of an interprofessional team, the importance of communication within a team, and the need for clarifications of expectations. Themes related to program structure were the need to provide learning from the perspective of different professions, the need to monitor the value of ongoing reflective practice activities, and the challenges of scheduling HPTs with different schedules and responsibilities. Themes related to clinical knowledge and competence were increased confidence in delivering care in an interprofessional setting and a better understanding of how to best manage clinically and socially complex patients. The fewest comments were provided on sustained relationships and shared decision making.

## 4. Discussion

Overall, HPT ratings of the four core domains, system impacts, and program practices were generally high at the baseline (AY16-17) and increased over time for most areas of the program, although the magnitude of these changes varied by domain and profession. The greatest increases were observed across all professions in sustained relationships. The largest increase in interprofessional collaboration was reported by physician residents, while nurse practitioner HPTs reported a decline. When compared to other groups, nurse practitioner HPTs also reported a decline in scores related to performance improvement, program practice, and shared decision making, while improvements were observed for physician residents for these domains. For associated health HPTs, the greatest increase in scores over time were related to performance improvement and system impact. Associated health HPTs had the highest ratings of performance improvement during the 3-year period when compared to other HPTs.

Qualitative results provided further evidence that HPTs valued the interprofessional training provided by the CoEPCE program. HPTs highlighted the importance of trust among an interprofessional primary care team and understanding all HPTs role on the primary care team, yet acknowledged the challenges associated with delivering care in an interprofessional primary care setting. Shared decision making with the patient and their families was acknowledged as critical to delivering patient-centered care, and ensuring the continuity of the interprofessional healthcare team is important for maintaining sustained relationships. They also identified areas where more advanced interprofessional training was needed for all HPTs, such as performance improvement, as well as suggested changes for program structure, especially for associated health professionals.

Overall high scores and positive qualitative feedback across all professions were not surprising due to the rigorous curriculum development and implementation process that occurred prior to the full implementation of the program during the baseline year AY16-17. In AY2011, CoEPCE curricula began with a focus on physician residents and nurse practitioner HPTs. During AY12-15, the curricula was refined and expanded to include specific training curriculum for associated health HPTs. For AY16-17, the CoEPCE curricula was finalized for all HPTs across all sites.

However, declines in score were observed over time, especially for nurse practitioner HPTs, as well as associated health HPTs. For instance, these HPTs would have had prior nursing practice in interprofessional collaboration before entering the CoEPCE program. Nurse practitioners have prior registered nurse experience in which interprofessional practice is common. Their educational trajectory is different from physicians in that they have undergraduate education and training as a registered nurse prior to attending graduate school for their nurse practitioner education. Therefore, their exposure to the interprofessional team is more in depth from an educational and experiential viewpoint. Therefore, they would be starting the program at a higher baseline level as compared to other HPTs, and their potential for increasing knowledge in this domain would be limited. Another potential explanation that may have occurred over time is the Dunning–Kruger effect, where HPTs develop sophisticated expectations about the curriculum over time that are not addressed by the advancement of the curriculum [19]. This could explain the decline in the scores reported by nurse practitioner HPTs in the domain of interprofessional collaboration, and the decline in scores for performance improvement among associated HPTs. Both of these HPT groups likely receive extensive training in these domains prior to entering the CoEPCE program, so more advanced curricula were needed for these HPTs as compared to physician residents. Another possible explanation that may influence HPTs perceptions of the curriculum are differences in HPTs’ professional goals and baseline profession-specific identity [20]. For example, the emphasis on performance improvement during pharmacy training may lead to pharmacy HPTs perceiving this part of the curriculum as more valuable when compared to other components of the curriculum. These potential issues can influence HPTs perception of interprofessional curricula and support the need for the careful consideration of these factors when designing interprofessional programs that include HPTs from various professions [21,22]. Addressing these factors is critical for designing an interprofessional curriculum that teaches HPTs to shift from delivering care in professional silos to team-based patient centered care. 

Changes in healthcare delivery emphasizing interprofessional teams requires interprofessional education that teach healthcare professionals to deliver team-based care. Delivering high-quality patient care requires the coordination and collaboration of providers across health professions. Yet, health profession education traditionally occurs in silos [23]. Previous studies have demonstrated the benefit of moving students out of educational silos into an interprofessional model, including understanding the value of other health professions, an increased emphasis on healthcare quality, and a positive attitude toward collaboration [20,24]. In the CoEPCE program, nurse practitioner residents, pharmacy residents, and psychology fellows were all post-graduate trainees, and likely had previous training in interprofessional healthcare delivery. Skills related to interprofessional practice among these trainees were likely developed over time and learned from previous clinical experience. Given these variations in training experiences and exposure, it can be difficult to adequately meet the needs of HPTs from different professions while developing and implementing interprofessional curricula based on common core domains.

Significant challenges of developing interprofessional curricula include identifying the clinical content relevant to all health professions and implementing teaching strategies that satisfy all HPTs. We found that psychologists reported being less satisfied in part because the clinical topics were not focused on mental health, but rather focused on topics more relevant to physician residents and nurse practitioner HPTs. The emphasis on clinical content that was more aligned with the needs of physician residents and nurse practitioner HPTs was likely an artifact of pharmacists and psychologists not being mandated to participate in the early phases of the program, and, although those HPTs could participate based on site discretion, the curriculum did not consistently incorporate those HPT needs. As the program expanded HPT professions, the pharmacy and psychology faculties were added to the program to provide mentoring to these HPTs; however, the revision of the core curricula to include clinical content specific for these HPTs took longer to implement.

Interprofessional curricula is designed to challenge professional values, and requires a significant shift in the way that healthcare professionals traditionally learn and practice [25]. Interprofessional curricula require cultural changes across professions that occur in stages and develop over time [25,26]. In this project, we wanted to understand how HPTs across various professions react to a common interprofessional curriculum based on a predetermined curriculum model that included the four core domains that were delivered using similar content and teaching methodology to all health professions. Teaching strategies were developed by interprofessional faculty, and attempts were made to integrate clinical content from different health professions into training activities. For example, the interprofessional skills curriculum was developed through collaboration across multiple training sites to teach HPTs how to incorporate the opinion of different healthcare professions into the care of the patient. Curricular developments over time were also influenced by numerous factors at the macro (systems), meso (organizational), and micro (clinic, faculty and learners) levels that continually influenced the developmental process and curriculum structure [27]. Lastly, increased attention has been given to IPE during recent years. However, the CoEPCE program was different from other interprofessional education programs in that the CoEPCE program was intervening in the clinical training/graduate space, rather than being limited to didactic learning. The vast majority of interprofessional education programs continue to be limited to didactic learning.

There were some challenges associated with implementing this program. First, the number of trainees from some of the health professions was low, which limited our ability to use quantitative strategies to assess program implementation in these groups. Second, subtle local curricular changes that occurred over time as the program expanded to include new health professions were difficult to track. The impact of specific programmatic changes that were implemented for specific health professions could not be assessed. In addition, local programmatic changes were also made based on the clinical environment and needs of the patient population, which may limit generalizability to other systems. However, the CoEPCE program was not designed as a trial to prove that the intervention was broadly applicable. It was a demonstration project to show that some intervention could move the needle in a large health system in a variety of settings based on a standard framework. Furthermore, there were differences in academic programs and participants that could not be accounted for in our analysis. Despite the challenges related to program implementation, graduates of the CoEPCE program reported overall high levels of satisfaction with the program, and valued the interprofessional skills training they received as part of this program [28].

There are several strengths of this work. First, this program offered a unique and generalizable curriculum that was intended to meet the needs of multiple health professions. Second, multiple trainings sites with highly diverse clinical populations participated in this program. Third, the generalizability of HPT perceptions about the program is also high, as multiple years of data from HPTs at multiple sites was examined. Lastly, many of the HPTs received part of their clinical training experience at non-VA sites, which could have led to potential program impacts beyond the VA.

In summary, the delivery of high-quality interprofessional team-based care is predicated on the development of effective interprofessional teaching strategies. Our findings support the need for the careful and deliberate consideration of profession-specific identity characteristics when designing interprofessional curricula. Future efforts are needed to broaden the implementation of interprofessional education to reduce silos among health professionals and improve the delivery of patient care.

## 5. Conclusions

The CoEPCE program is the first national interprofessional training program that included HPTs from various disciplines across the US. This program was unique in that all CoEPCE program sites were provided with a standardized curricular framework, but were encouraged to personalize their curriculum based on their local environment and patient population. As the CoEPCE curricula and delivery was refined at both the national and local levels, we observed improvements in trainee perceptions over time. Although national funding for the national program has ended, several local sites continue to deliver the CoEPCE curriculum, as well as having expanded their previous efforts to better meet the needs of HPTs.

## Figures and Tables

**Table 1:** Comparison by profession at baseline academic year 2016–2017.

	Medicine			Nursing			Associated Health			
	n	Mean (SD)	Median (IQR)	n	Mean (SD)	Median (IQR)	n	Mean (SD)	Median (IQR)	*p*-Value *
Inter-Professional Collaboration	65	4.2 (0.7)	4.3 (1.3)	33	4.3 (0.6)	4.3 (0.8)	42	4.3 (0.6)	4.4 (1.0)	0.78
Performance Improvement	65	3.7 (0.9)	4.0 (0.8)	33	3.9 (0.8)	4.0 (1.3)	42	3.7 (0.8)	3.5 (1.0)	0.57
Program Practices	65	4.1 (0.7)	4.0 (0.9)	33	4.3 (0.7)	4.4 (1.1)	42	4.0 (0.7)	3.9 (1.0)	0.09
Shared Decision Making	65	4.0 (0.7)	4.0 (1.0)	33	4.2 (0.6)	4.2 (0.8)	42	3.9 (0.6)	4.0 (0.6)	0.22
Sustained Relationships	65	4.1 (0.7)	4.0 (1.0)	33	4.3 (0.6)	4.3 (1.0)	42	3.9 (0.7)	4.0 (0.5)	0.08
System Impact	65	4.4 (0.6)	4.3 (1.0)	33	4.6 (0.5)	4.8 (0.6)	42	4.1 (0.6)	4.1 (0.9)	0.0013

* Kruskal–Wallis test.

**Table 2:** Summary of qualitative feedback from HPTs on the CoEPCE four curricular domains and key program components.

Component	Number of Comments	Themes	Quotes
CoEPCE Four Curricular Domains			
Shared Decision Making	43	Using motivational interviewing to facilitate behavior change Use of a team approach to facilitate healthcare planning	“Motivational interviewing techniques were useful for supporting and encouraging patients to quit smoking.” “Shared decision making is important to decide best outcomes for the patient.”
Sustained Relationships	29	Building relationships with other interprofessional team members Value of continuity of care for patients	“This was an opportunity to develop relationships with HPTs from other disciplines.” “I have also learned the value of a self-contained system that provides patients with continuity of care.”
Interprofessional Collaboration	418	Understanding the training and background of other health professionals Importance of trust among all healthcare providers Challenges of working as part of an interdisciplinary care team Importance of communication within an interprofessional team Need for clarification of expectations in clinical settings	“It is important to know what other health professions can bring to help with medical problems and patient’s concerns.” “That true interprofessionalism only occurs when the players involved trust and understand each other as providers and people.” “Model of interprofessional care works well, but still has growing pains and people are actively trying to adapt and sort out the best way to deliver care in an interprofessional and interdisciplinary environment.” “I learned different ways to engage in interprofessional collaboration (consultation, warm handoffs, e-consults, co-visits).” “More clearly defined expectations for what my role is as a resident on the team and what my team members should be doing for each patient.”
Performance Improvement	113	Importance of panel management in improving clinical care Challenges associated with conducting QI Research Importance of data and use of technology to facilitate improvement in clinical care Quality improvement integration into everyday practice	“More teaching is needed regarding panel management.” “I really value learning more about QI/PI projects when we’re given enough time/resources to engage and follow through with them.” “Additional training in panel management and using analytical tools.” “I also learned a bit about what QI looks like in a busy clinical setting.”
Key Program Components			
Clinical Knowledge and Competence	227	Interest in more VA-specific clinical training Confidence in delivering care in a primary care setting Managing clinically and socially complex patients	“Having more electives/curriculum specific to the VA, e.g., women’s health, transgender health, PTSD care.” “How to manage patients in an outpatient setting. I feel comfortable being a primary care doctor after graduating from COE.” “Include more social determinants of health, and how to effectively address them from a clinical context.”
Program Structure	325	Providing didactic learning from the perspective of different professions Monitor value of ongoing reflective practice activities Challenges of scheduling providers for interprofessional clinics Barriers to scheduling HPTs to attend group didactic sessions due to competing responsibilities	“More pharmacy-related topics within existing didactics (i.e., discussing the medications, not just the diagnosis).” “Some of the reflective practices became redundant toward the attend and turned into more of an “assignment” than a growing and development opportunity.” “Would like more opportunities to interact with HPTs of other disciplines in the clinical setting, not just during didactics.” “Create an outline/syllabus/calendar that is easily accessible for HPTs to use so they can better understand what didactics are happening when.”
Professional Development	90	Learning time management skills Need for more structured mentoring Transition to a career in interprofessional primary care	“I learned how to balance a variety of responsibilities.” “More formal mentorship with allocated time.” “My training has helped to affirm that I would like to practice within an interprofessional patient aligned care team.”

## Data Availability

Data are contained within the article.
